# Galectin-1 reduces the severity of dextran sulfate sodium (DSS)-induced ulcerative colitis by suppressing inflammatory and oxidative stress response

**DOI:** 10.17305/bjbms.2019.4539

**Published:** 2020-08

**Authors:** Pelin Arda-Pirincci, Guliz Aykol-Celik

**Affiliations:** 1 Department of Biology, Faculty of Science, Istanbul University, Istanbul, Turkey; 2 Institute of Graduate Studies in Sciences, Section of Biology, Istanbul University, Istanbul, Turkey

**Keywords:** Galectin-1, dextran sulfate sodium (DSS), ulcerative colitis, inflammation, oxidative stress

## Abstract

Ulcerative colitis is an inflammatory bowel disease that affects a large number of people around the world. Galectin-1 is a β-galactoside-binding lectin with a broad range of biological activities. The effects of galectin-1 on dextran sulfate sodium (DSS)-induced ulcerative colitis in vivo is not clear. We investigated the effect of galectin-1 on colon morphology, cell proliferation, oxidative stress, antioxidant system, and proinflammatory/antiinflammatory cytokines in a DSS-induced mouse model of ulcerative colitis. Thirty-two C57BL/6 mice were randomly assigned to one of the four groups: control, acute colitis, galectin-1, and DSS+galectin-1. Controls were treated with phosphate-buffered saline (PBS) for seven days. Acute colitis was induced by 3% DSS in drinking water administered orally for five days. Mice in galectin-1 groups were treated with 1 mg/kg recombinant human galectin-1 in PBS for seven consecutive days. Oral DSS administration resulted in acute colitis by causing histopathological changes; an increase in disease activity index (DAI), lipid peroxidation (malondialdehyde [MDA]), myeloperoxidase (MPO), and tumor necrosis factor (TNF)-α levels; a decrease in body weight, colon length, cell proliferation index, catalase, glutathione peroxidase (GSH-Px) and superoxide dismutase (SOD) activities, and GSH and interleukin (IL)-10 levels. The treatment with galectin-1 attenuated DSS-induced acute colitis by reducing DAI, MDA, MPO, and TNF-α levels and by increasing body weight, colon length, cell proliferation, antioxidant enzyme activity, GSH, and IL-10 levels. These findings suggest that galectin-1 has proliferative, antioxidant, antiinflammatory, and cytoprotective effects against DSS-induced ulcerative colitis in mice. Due to its antiinflammatory and antioxidant activity galectin-1 may be effective in preventing and treating ulcerative colitis.

## INTRODUCTION

Ulcerative colitis is characterized by severe inflammation and ulcer formation in the colon mucosa and submucosa. It can be caused by genetic factors or various infectious agents. In addition, ulcerative colitis can be triggered by factors such as smoking, stress, alcohol, and refined foods [1]. Many studies have been carried out to understand the underlying mechanism of ulcerative colitis [2,3], and the potential mechanisms include infiltration of inflammatory cells, activation of T cells, induction of proinflammatory cytokines, and oxidative stress. The current drugs used in ulcerative colitis therapy, immunosuppressive agents and antiinflammatory drugs, only eliminate inflammation and do not eliminate the root causes of colitis [4,5]. Therefore, new and effective treatment methods for ulcerative colitis need to be developed.

*In vivo* animal models generated using chemical agents such as dextran sulphate sodium (DSS), trinitrobenzenesulfonic acid (TNBS), indomethacin, acetic acid, iodoacetamide, and oxazolone have not only been helpful for investigating the pathogenesis of colitis, but have also facilitated the development of more effective treatment methods [6]. Moreover, the ulcerative colitis model induced with DSS has advantages compared to the other models, because DSS-induced colitis in rodents is quite similar to human colitis, in terms of clinical and histopathological features [7,8].

Galectin-1 (Gal-1) is a homodimeric lectin that belongs to the galectin family and has the affinity for β-galactoside [9]. Gal-1 has a role in cell growth, proliferation, differentiation, adhesion, migration, T cell apoptosis, and immunomodulation. It participates in the regulation of wound healing, angiogenesis, and antiinflammatory response [10]. In recent years, Gal-1 has been tested in experimental animals for the treatment of various diseases, including autoimmune encephalomyelitis [11], arthritis [12], hepatitis [13], and pancreatitis [14]. Nevertheless, the use of Gal-1 in *in vivo* models is still very limited and the mechanisms of action have not been fully elucidated.

To the best of our knowledge, no study has investigated the effects of Gal-1 administration on DSS-induced ulcerative colitis in experimental animals. The aim of this study was to investigate the effect of Gal-1 on colon morphology, cell proliferation, oxidative stress, antioxidant system, and proinflammatory/antiinflammatory cytokines in a DSS-induced mouse model of ulcerative colitis. In addition, this study aimed to explore the prophylactic and therapeutic benefits of Gal-1 in ulcerative colitis.

## MATERIALS AND METHODS

### Animal model

**FIGURE 1:**
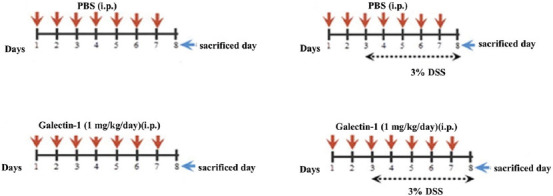
Schematic diagram of the experimental design. Group I: control mice; Group II: mice induced with DSS; Group III: mice injected with galectin-1; Group IV: mice administered DSS and galectin-1. DSS: Dextran sulfate sodium.

The Animal Care and Use Committee of Istanbul University approved the experimental protocol of this study with the number 2012/95. Thirty-two C57BL/6 mice, 7–8 weeks old, were randomly selected and separated into four groups. The schematic diagram of the experimental design is shown Figure 1. The following groups were included: group I, control animals were injected intraperitoneally (i.p.) with phosphate-buffered saline (PBS, pH 7.4) once a day for 7 consecutive days; group II, animals were orally dosed 3% DSS (w/v, mol. wt. 36,000–50,000) in their drinking water for 5 days for acute colitis induction; group III, animals were injected i.p. with 1 mg/kg Gal-1 (dissolved in PBS) once a day for 7 consecutive days; and group IV, animals were injected i.p. with 1 mg/kg Gal-1 for 7 sequential days and received 3% DSS orally for 5 days (starting on the 3^rd^ day of Gal-1 treatment). The animals were fasted overnight prior to euthanization. The animals were euthanized under anesthesia with ketamine, on the 8^th^ day of the experiment. The colon tissues were removed from the animals for examination.

### Determination of the change in body weight and colon length of mice

The weights of mice were monitored daily during the experiment, to assess the change in the body weight. The mouse body weight before starting the experiment was determined to calculate the percentage change in body weight. Midline incisions of anaesthetized mice were performed. The colon tissues from the cecum to the anus were collected and the fecal content was carefully removed before measuring the colon length.

### Colitis activity index

The Cooper’s grading system for the degree of colonic inflammation was used and the disease activity index (DAI) was determined for DSS-induced mice [15]. The DAI scoring criteria, shown in Table 1, included occult/gross rectal bleeding, stool consistency, and weight loss. Each DAI parameter was scored between 0 (undamaged) and 4 (severe damage) and the arithmetic average was taken. The maximum score for an individual animal was 4 and mice with a score ≥3 were used.

**TABLE 1:**
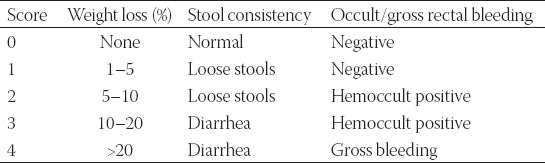
Disease activity index scoring criteria

### Histopathological evaluation

Tissues obtained from the first 0.5-cm segments of the proximal part of the distal colon were fixed in Bouin solution and embedded in paraffin. The colon sections (5 µm thick) were stained with Periodic acid-Schiff (PAS) and hematoxylin-eosin (HE), viewed under a light microscope (Olympus CX41, Japan), and pictures were taken with a digital camera (Olympus DP71). The colitis scoring system was used for ***microscopic*** determination of histological colitis damage score in distal colon sections [16]. Colon sections were graded based on the following criteria: severity of inflammation, spread of inflammation, and crypt damage (Table 2). Subsequently, the score for each criterion was multiplied by the percentage involvement on each cross-section and the calculated scores of the three criteria were added. The maximum colitis damage score was accepted as 40 for an individual animal and individual colitis scores were statistically evaluated.

**TABLE 2:**
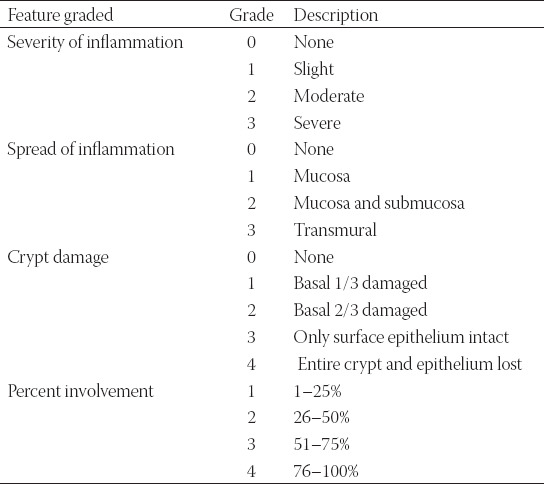
Histological colitis damage scoring system

### Immunohistochemical analysis

Cell proliferation indexes of distal colon mucosa were assayed using Ki-67 immunohistochemistry. Colon samples were fixed in 10% phosphate-buffered formalin and embedded in paraffin. Mouse anti-Ki-67 antibody (Abcam-ab16667, Cambridge, UK; 1:100 in PBS) was used to detect dividing cells. Mayer’s hematoxylin was applied as contrasting dye. In colon sections, approximately 1000 cells in different randomly selected areas were counted. The proliferation index was calculated as a percentage of Ki-67 positive cells relative to the total cell number.

### Biochemical analysis

Colon homogenates (10%) were prepared in cold 0.9% sodium chloride solution. They were centrifuged at 10,000 g for 20 minutes and the supernatants were used for spectrophotometric analysis. Protein levels were estimated according to Lowry protocol using bovine serum albumin (BSA) standards [17]. The Beutler method was used for determination of glutathione (GSH) level in colon homogenates [18]. Malondialdehyde (MDA), which is an end product of lipid peroxidation, was assayed according to the Ledwozyw method [19]. Spectrophotometrically (Schimadzu UV 1700, Kyoto, Japan), glutathione peroxidase (GSH-Px) activity was assayed according to the Paglia-Valentine method [20], catalase (CAT) activity according to the Aebi method [21], and superoxide dismutase (SOD) activity according to Sun method [22].

### Enzyme-linked immunosorbent assay (ELISA)

Mononuclear cell infiltration into colon tissue was detected by measuring myeloperoxidase (MPO) concentration. Colon tissues (10 mg) were homogenized in 200 µl of lysis buffer (pH 7.4) with a glass homogenizer. Subsequently, samples were centrifuged at 1500 g for 15 minutes at +4°C and the supernatants were used to determine MPO levels in colon tissues. Measurement was done according to the instructions of Mouse MPO Sandwich ELISA Kit (HK210, Hycult Biotech, Uden, Netherlands).

Tumor necrosis factor alpha (TNF-α) and interleukin (IL)-10 expression in colon tissues were determined by ELISA. Colon tissues were homogenized in a glass homogenizer by diluting with ice-cold PBS (0.01 M, pH 7.2) at 10%. Then, homogenates were subjected to ultrasonication 6 times for 10 seconds, to lyse the cell membrane. Next, homogenates were centrifuged at 5000 g at +4°C for 5 minutes, and clear supernatants were used in cytokine and protein measurements. Following the manufacturer’s recommendations, TNF-α levels were assayed using mouse specific TNF-α Sandwich ELISA Kit (SEA133Mu, USCN, Cloud-Clone Corp., Wuhan, China), while IL-10 concentrations in colon tissues were measured using Mouse IL-10 Sandwich ELISA Kit (SEA056Mu, USCN, Cloud-Clone Corp., Wuhan, China). Protein levels in supernatants were determined by the Lowry’s method for ELISA analysis.

### Statistical analysis

All data were evaluated using SPSS for Windows, Version 15.0. (SPSS Inc., Chicago, USA). The colitis activity index, histological colitis score, and immunohistochemical data were analyzed by Kruskal–Wallis test and Mann–Whitney U test. All other data were evaluated using one-way ANOVA and unpaired student t-test. The results were represented as mean ± standard error (SE).

## RESULTS

### Relative change in body weight and colon length

**FIGURE 2:**
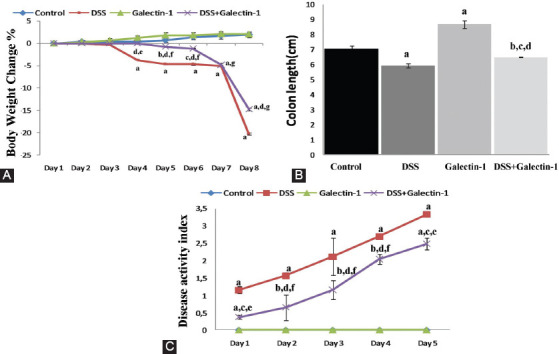
(A) Body weight change (%) in all groups. Beginning from the 4^th^ day of the experiment, when the symptoms of colitis were observed, a significant decrease in the body weight of mice in DSS group was detected compared to control group. The body weight of mice in DSS+galectin-1 group significantly increased throughout the experiment from the 4^th^ day (except on the 7^th^ day) compared to DSS group. ^a^*p* < 0.001, ^b^*p* < 0.05, and ^c^*p* < 0.01 vs. control group; ^d^*p* < 0.001 vs. DSS group; ^e^*p* < 0.05, ^f^*p* < 0.01, and ^g^*p* < 0.001 vs. galectin-1 group. (B) Colon length in all groups. The lengths of colon tissues in DSS group were markedly decreased compared to control group. There was a remarkable increase in the colon lengths in the group treated with Gal-1 alone compared to control animals. The colon lengths in DSS+galectin-1 group were significantly increased compared to DSS group. ^a^*p* < 0.001 and ^b^*p* < 0.05 vs. control group; ^c^*p* < 0.01 vs. DSS group; ^d^*p* < 0.001 vs. galectin-1 group. (C) Daily disease activity index (DAI) in DSS-induced experimental groups. DAI was measured at 24, 48, 72, 96, and 120 hours after DSS administration. DAI was elevated with the induction of ulcerative colitis. Gal-1 treatment significantly reduced DAI compared to DSS group. ^a^*p* < 0.001 and ^b^*p* < 0.01 vs. control group; ^c^*p* < 0.001 and ^d^*p* < 0.01 vs. DSS group; ^e^*p* < 0.001 and ^f^*p* < 0.01 vs. galectin-1 group. DSS: Dextran sulfate sodium.

The relative change in the body weight of mice from different groups is shown in Figure 2A. In the first 3 days of the experiment, no significant difference was detected among the groups in terms of the change of the body weight. After 7 days, a mild increase in the body weight of mice in control and galectin-1 group was observed. Beginning from the 4^th^ day of the experiment, when the symptoms of colitis were observed, a significant decrease in the body weight of mice in DSS group was detected compared to control group (*p* < 0.001). The body weight of mice in DSS+galectin-1 group significantly increased throughout the experiment from the 4^th^ day (except on the 7^th^ day) compared to DSS group (*p* < 0.001).

The colon lengths of mice from each group are shown in Figure 2B. There was a statistically significant decrease in the length of colon tissues in DSS group compared to control group (*p* < 0.001). However, a significant increase was observed in galectin-1 group compared to control group (*p* < 0.001). In DSS+galectin-1 group, the colon length was decreased compared to control (*p* < 0.05) and galectin-1 group (*p* < 0.001). On the other hand, the colon length was significantly increased in DSS+galectin-1 group compared to DSS group (*p* < 0.01).

### Colitis activity index

The DAI of different groups determined for 5 days following DSS administration is presented in Figure 2C. DAI was found to be elevated with the induction of ulcerative colitis. The highest DAI values in both DSS group and DSS+galectin-1 group were observed on the 5^th^ day of DSS administration. DSS-induced mice exhibited significantly higher DAI levels compared to control mice (*p* < 0.001). In DSS+galectin-1 group, there was a significant increase in DAI compared to control and galectin-1 group (day 1 and 5 *p* < 0.001; and day 4 *p* < 0.01). However, DAI in DSS+galectin-1 group was lower than in DSS group at all experimental days (day 1 and 5 *p* < 0.001; and day 4 *p* < 0.01).

### Histopathological results

**FIGURE 3:**
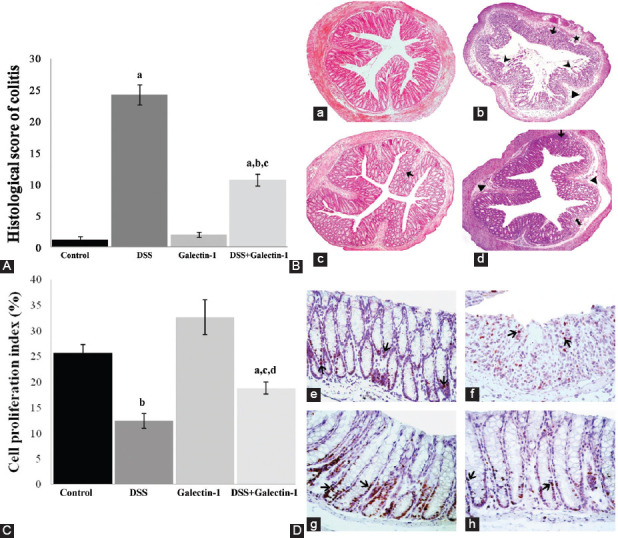
(A) Histological colitis score of all groups. The histological score showed a statistically significant increase in DSS compared to control group. The pre-injection of Gal-1 to DSS-induced mice significantly reduced the colonic damage. Data are given as mean ± SE per group. ^a^*p* < 0.001 vs. control group; ^b^*p* < 0.001 vs. DSS group; ^c^*p* < 0.001 vs. galectin-1 group. (B) Light microscope image of the colon in control group (a). Light micrograph of DSS-induced ulcerative colitis group (b) shows inflammatory cell infiltration (⟶), submucosal edema (►), focal mucosal deterioration (★), necrotic areas in the mucosa and submucosa (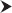), disruption of crypt integrity and widespread loss of crypts. Galectin-1 group (c) and DSS+galectin-1 group (d) micrographs show slight inflammatory cell infiltration (⟶). In addition, the image of DSS+galectin-1 group demonstrates submucosal edema (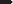) and disruption of crypt integrity (►). Hematoxylin-eosin; original magnification ×100. (C) Cell proliferation index determined by Ki-67 immunohistochemistry in colon mucosa. The cell proliferation index of DSS group showed a statistically significant decrease compared to control group. The suppressive effects of DSS on the proliferation of colon epithelial cells were inhibited by Gal-1 pre-injection, and the proliferation index markedly increased compared to DSS group. ^a^*p* < 0.05 vs. control group; ^b^*p* < 0.01 vs. control group; ^c^*p* < 0.05 vs. DSS group; ^d^*p* < 0.01 vs. galectin-1 group. (D) Proliferating cells marked by Ki-67 immunohistochemistry in the distal colon mucosa of mice (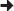). Control group (e), DSS group (f), galectin-1 group (g), DSS+galectin-1 group (h). Streptavidin-biotin-peroxidase staining method; original magnification ×400. DSS: Dextran sulfate sodium.

The histological colitis damage score and light microscope images of the colon from all groups are presented in Figure 3A and B. The histological score showed a statistically significant increase in DSS compared to control group (*p* < 0.001). No significant difference was found in the histological score between mice in galectin-1 group and mice in control group. The histological damage score was significantly increased in DSS+galectin-1 group compared to both control and galectin-1 groups (*p* < 0.001). However the pre-injection of Gal-1 to DSS group significantly decreased histological damage compared to DSS group (*p* < 0.001). The sections taken from the distal colon tissues of mice from control group showed healthy and normal histological appearance. The most obvious findings in DSS group were extensive inflammatory cell infiltration in the mucosa and submucosa, submucosal edema, focal mucosal deterioration, superficial erosion in epithelium, disruption of crypt integrity, vacuolar hydropic degeneration in crypt cells of some individuals, widespread ulceration with loss of crypts, and necrotic areas in mucosa and submucosa. The histological appearance in the tissue sections of galectin-1 group was similar to that of control group, except for mild inflammatory cell infiltration observed in the lamina propria of a few individuals. The histological damage in DSS+galectin-1 group was markedly reduced compared to DSS group. Mucosal ulceration, necrotic areas, and severe crypt lesions were not observed in the sections of DSS+galectin-1 group. The pre-injection of Gal-1 to DSS-induced mice significantly reduced the colonic damage by preventing necrotic damage and preserving the mucosal structure in the distal colon.

### Immunohistochemical results

Figure 3C shows cell proliferation index and Figure 3D shows Ki-67 immunoreactive micrographs of colon mucosa in all groups. The cell proliferation index of DSS group showed a statistically significant decrease compared to control group (*p* < 0.01). A statistically significant increase in cell proliferation index in galectin-1 group was observed compared to control group. In DSS+galectin-1 group, the number of Ki-67 positive cells was significantly decreased (*p* < 0.05, *p* < 0.01) compared to control and galectin-1 group, but a significant increase in cell proliferation compared to DSS group was noticed (*p* < 0.05).

### Biochemical results

#### GSH and lipid peroxidation levels

**FIGURE 4:**
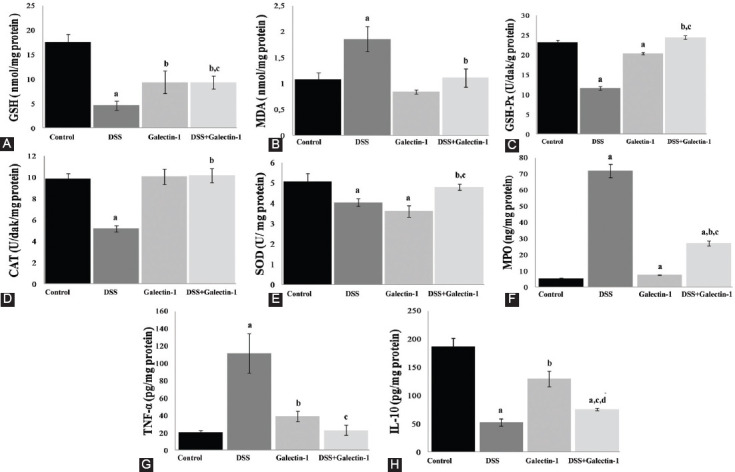
(A) GSH levels in the mouse colon tissues. ^a^*p* < 0.001 and ^b^*p* < 0.01 vs. control group; ^c^*p* < 0.05 vs. DSS group. (B) MDA levels in the mouse colon tissues. ^a^*p* < 0.05 vs. control group; ^b^*p* < 0.05 vs. DSS group. (C and D) GSH-Px and CAT activities in the mouse colon homogenates. ^a^*p* < 0.001 vs. control group; ^b^*p* < 0.001 vs. DSS group; ^c^*p* < 0.001 vs. galectin-1 group. (E) SOD activity in the mouse colon tissues. ^a^*p* < 0.05 vs. control group; ^b^*p* < 0.05 vs. DSS group; ^c^*p* < 0.01 vs. galectin-1 group. (F) MPO concentration in the mouse colon homogenates. ^a^*p* < 0.001 vs. control group; ^b^*p* < 0.001 vs. DSS group; ^c^*p* < 0.001 vs. galectin-1 group. (G) TNF-α levels in the mouse colon tissues. ^a^*p* < 0.01 vs. control group; ^b^*p* < 0.05 vs. control group; ^c^*p* < 0.01 vs. DSS group. (H) IL-10 cytokine levels in the mouse colon tissues. ^a^*p* < 0.001 vs. control group; ^b^*p* < 0.05 vs. control group; ^c^*p* < 0.05 vs. DSS group; ^d^*p* < 0.01 vs. galectin-1 group. All data are given as mean ± SE per group. GSH: Glutathione; DSS: Dextran sulphate sodium; MDA: Malondialdehyde; GSH-Px: Glutathione peroxidase; CAT: Catalase; SOD: Superoxide dismutase; MPO: Myeloperoxidase; TNF-α: Tumor necrosis factor alpha; IL: Interleukin.

The GSH and lipid peroxidation (MDA) levels of colon homogenates from all groups are shown in Figure 4A and B. GSH levels in all experimental groups showed a statistically significant decrease compared to control group; the significance of the difference was as follows: DSS (*p* < 0.001), galectin-1 (*p* < 0.01), and DSS+galectin-1 (*p* < 0.01). The most significant reduction in GSH levels was in DSS group. The GSH level in DSS+galectin-1 group significantly increased compared to DSS group (*p* < 0.05). MDA levels in DSS group showed a significant increase compared to control group (*p* < 0.05). Compared to control animals, an insignificant decrease was observed in galectin-1 group. The MDA level in DSS+galectin-1 group significantly decreased compared to DSS group (*p* < 0.05).

#### Antioxidant enzyme activities

GSH-Px, CAT, and SOD activities in colon tissues from all groups are presented in Figure 4C, D, and E, respectively. GSH-Px activity was significantly decreased in both DSS and galectin-1 group compared to control group (*p* < 0.001). DSS administration reduced GSH-Px activity by half compared to control animals. GSH-Px activity was significantly increased in DSS+galectin-1 group compared to DSS and galectin-1 group (*p* < 0.001). CAT activity in DSS group showed a significant decrease compared to control group (*p* < 0.001). However, Gal-1 pre-injection to DSS group caused a statistically significant increase in CAT activity compared to DSS group (*p* < 0.001). SOD activity was significantly decreased in both DSS and galectin-1 group when compared to control group (*p* < 0.05). The low SOD activity observed in DSS group significantly increased with the pre-injection of Gal-1 (*p* < 0.05). In addition, the increase in SOD activity observed in DSS+galectin-1 group was statistically significant compared to galectin-1 group (*p* < 0.01).

#### MPO and cytokine levels

The MPO concentration of colon homogenates from all groups is presented in Figure 4F. The most significant increase in MPO concentration (about 13-fold) was observed in DSS group compared to control group (*p* < 0.001). In addition, there was a statistically significant increase in MPO concentration in both galectin-1 and DSS+galectin-1 group (*p* < 0.001) compared to control. Furthermore, a significant increase in MPO concentration was detected in DSS+galectin-1 compared to galectin-1 group (*p* < 0.001). But MPO concentration in DSS+galectin-1 group was significantly lower compared to DSS group (*p* < 0.001).

Figure 4G and H show TNF-α and IL-10 cytokine levels of colon tissues from all groups. There was a statistically significant increase in TNF-α levels in both DSS (*p* < 0.01) and galectin-1 group (*p* < 0.05) compared to control group. The pre-injection of Gal-1 to DSS-induced mice significantly increased TNF-α levels (*p* < 0.01). There was a statistically significant decrease in IL-10 levels in DSS group compared to control group (*p* < 0.001). Also, a significant decrease in IL-10 levels in galectin-1 (*p* < 0.05) and DSS+galectin-1 group (*p* < 0.001) was observed compared to control group. However the pre-injection of Gal-1 to DSS-induced mice increased IL-10 levels compared to DSS group (*p* < 0.05). On the other hand, IL-10 levels were decreased in DSS+galectin-1 group compared to galectin-1 group (*p* < 0.01).

## DISCUSSION

DSS-induced mouse ulcerative colitis model is commonly used and is the preferred experimental model for ulcerative colitis. This is because this model shows clinical features such as weight loss, diarrhea, rectal bleeding, as well as histopathological features such as inflammatory cell infiltration, ulceration, and crypt damage of the colon mucosa. Acute or chronic colitis models can be generated by applying different doses of DSS to experimental animals [23-25].

In several studies, oral administration of DSS solution (3%) to mice for 5 days resulted in severe colitis damage characterized by bloody diarrhea, constant weight loss, increased DAI, and decreased colon length. Moreover, colon sections of DSS-induced mice exhibit ulcerated areas, disintegrated crypt structure, inflammatory cell infiltration, and severe microscopic damage [26,27]. These findings are consistent with the results of our study, where 3% DSS was applied for 5 days to C57BL/6 mice and DAI was >3 in all animals. The histopathological findings of the study by Matos et al. [27] showed inflammatory cell infiltration, mostly in the mucosa and in some cases in the submucosa, submucosal edema, disruption of crypt integrity, widespread ulceration, and necrosis with loss of crypts. The pathogenesis of ulcerative colitis is directly associated with the infiltration of a large number of leucocytes to the mucosa of the colon. It is well-known that neutrophil infiltration to the mucosa is one of the most important events in colon inflammation during the acute colitis phase. MPO activity, which is an indicator of neutrophil infiltration, was shown to increase with the severity of inflammatory damage as observed by microscopic analysis in many studies where DSS was used to induce ulcerative colitis in mice [28,29]. This is in agreement with the microscopic findings of the present study, where MPO concentration in colon tissues from DSS group significantly increased compared to control group.

Gal-1, a lectin expressed by different types of cells in the gastrointestinal tract of human and mouse, has a role in many biological processes, including wound healing and antiinflammatory response. Gal-1 is important for controlling the initiation, increase, and termination of inflammatory responses [9,30]. It also prevents migration of several types of inflammatory cells from peripheral blood and the extracellular matrix [31]. There is strong evidence suggesting that Gal-1 acts as an immunosuppressive and antiinflammatory mediator by inhibiting leukocyte aggregation. The inhibitory effect of Gal-1 on the accumulation of leucocytes has been demonstrated in various *in vivo* inflammation models, such as phospholipase A2-stimulated or carrageenan-stimulated paw edema [32] and acute peritonitis [33]. In phospholipase A2-stimulated paw edema, Gal-1 pre-treatment heals edema by decreasing polymorphonuclear leukocytes (PMN) infiltration and mast cell degranulation, thus reducing tissue damage [34]. This is consistent with our findings; 1 mg/kg dose of Gal-1 significantly reduced DAI and showed a protective effect on the colon by preventing the common inflammation, necrosis, and crypt damage caused by DSS. In addition, Gal-1 decreased MPO concentration in the colon tissue.

In ulcerative colitis, the balance between epithelial cell proliferation and apoptosis is impaired. Destruction of the epithelial barrier causes the invasion of microorganisms in the large intestine, leading to chronic inflammation of the colon mucosa [35]. In this study, DSS reduced epithelial cell proliferation in the mouse colon. Comparable to our findings, the rate of proliferating epithelial cells in crypts was significantly reduced compared to control group when Araki et al. administered DSS (5%, w/v) for 8 days to BALB/cAJcl mice [36]. These findings can be explained by the short-term DSS administration that arrests epithelial cells in the G0 phase and blocks the cell cycle, leading to mucosal damage. Recombinant Gal-1 shows biphasic effects on cellular proliferation depending on the concentration. Gal-1 is a mitogen at low concentrations, while the inhibitory effects of this lectin on proliferation occur at higher concentrations [37]. Gal-1 has a mitogenic effect on vascular smooth muscle cells [38], pulmonary artery endothelial cells [39], hepatic stellate cells [40], and mouse BALB3T3 fibroblast cells [41]. In the present study, the cell proliferation index in the colon mucosa of mice that received only Gal-1 injection was increased compared to control group. In addition, the suppressive effects of DSS on the proliferation of colon epithelial cells were inhibited by Gal-1 pre-injection, and the proliferation index markedly increased compared to DSS group. Furthermore, the colon lengths in DSS+galectin-1 group were significantly increased compared to DSS group. There was a remarkable increase in the colon lengths in the group treated with Gal-1 alone compared to control animals. These findings are consistent with the increase in the proliferation index of the colon mucosa in galectin-1 group. Thus, the effect of Gal-1 on the colon length may be associated with increased cell proliferation.

One of our aims was to determine the role of oxidative stress and antioxidant defense system in DSS-induced ulcerative colitis, and to investigate the effects of Gal-1 on oxidative damage caused by DSS in the colon tissue. Reactive oxygen metabolites, along with excessive production of proinflammatory mediators, may cause ulcerative colitis. Under pathological conditions, the balance between oxidants and antioxidants is disrupted [42]. In ulcerative colitis, active neutrophils that migrate to the colon mucosal epithelium cause oxidative damage and lipid peroxidation of colon tissue by producing reactive oxygen species (ROS) [43]. It was reported that after DSS administration ROS levels in colon mucosa significantly increase [44]. Several studies investigated the relationship between oxidative stress and antioxidant system in DSS-induced colitis models [3,28,29]. Yao et al. [3] and Zhao et al. [29] found that colon MPO and MDA levels increased, while SOD and GSH-Px activities decreased in colitis group compared to controls. In general, DSS increases oxidative stress and suppresses the antioxidant response in acute colitis models induced by 5% DSS. Our findings showed that MPO and MDA levels in the colon tissue significantly increased, while antioxidant levels markedly decreased in DSS-induced colitis group compared to control group. This is consistent with the studies cited above. These results suggest that, in DSS-induced ulcerative colitis model, oxidative stress and lipid peroxidation are increased in the colon tissue, due to the activation of neutrophils and macrophages, while the antioxidant defense system is greatly suppressed.

Gal-1 effect on oxidants and antioxidants in different tissues is not fully understood. In cancer cells, however, Gal-1 expression has been reported to increase under hypoxia and oxidative stress [45]. The present study is the first to reveal the effects of Gal-1 on MDA and GSH levels, and GSH-Px, CAT, and SOD activities. In this study, the pre-injection of Gal-1 to DSS-induced mice caused a statistically significant increase in GSH levels and GSH-Px, CAT, and SOD activities, and a significant decrease in MDA levels in the colon homogenates when compared to DSS group. Based on these findings, we presume that Gal-1 activates antioxidant defense system in DSS-induced ulcerative colitis model and protects colon tissue against oxidative damage.

Cytokines are important in the regulation of immune function. It is well-known that the balance between pro and antiinflammatory cytokines is impaired in inflammatory bowel diseases [46]. Colon biopsies of people with inflammatory bowel disease are reported to have increased proinflammatory cytokine levels and decreased antiinflammatory cytokine levels. DSS-induced ulcerative colitis model has shown that TNF-α plays an important role in the progression of colitis [47]. IL-10 is known to have antiinflammatory effects in inflammatory bowel diseases. IL-10 knock-out mice develop chronic colitis, which supports the view that IL-10 has an important role in immune regulation [48]. In a study where 3% DSS (40 kDa) was administered for 7 days to induce acute colitis in C57BL/6 mice, serum TNF-α levels were quite high compared to control group, while IL-10 levels decreased in the colitis group [49]. In the present study, TNF-α level showed a statistically significant increase in the colon homogenates of DSS group, whereas IL-10 levels decreased. In TNBS-stimulated colitis model, intravenous Gal-1 injection at different doses (0.04, 0.4, and 1 mg/kg) after intrarectal TNBS administration significantly decreased both plasma and colon proinflammatory cytokine levels, including TNF-α, IL-1β, IL-12, and interferon gamma (IFN-γ) in a dose-dependent manner [50]. No previous reports exist on the effect of Gal-1 on colon IL-10 levels. In our study, we detected a decrease in TNF-α level in DSS+galectin-1 group approaching the level observed in control group. In addition, the Gal-1 pre-treatment of DSS-induced mice significantly increased IL-10 levels compared to DSS-induced colitis group. These findings show that Gal-1 exerts antiinflammatory effect by decreasing elevated TNF-α level during inflammation and by increasing IL-10 level. Therefore, Gal-1 may play a protective role against ulcerative colon injury.

## CONCLUSION

In conclusion, this study suggests that Gal-1 has cytoprotective, proliferative, antioxidant, and antiinflammatory effects against experimental acute ulcerative colitis induced by DSS. The study is unique in a way that it was the first to examine the role of exogenously administered Gal-1 in DSS-induced experimental ulcerative colitis. Our results suggest that Gal-1 may be effective in preventing and treating ulcerative colitis due to its antiinflammatory and antioxidant function.
